# Transition of psychiatric care from childhood to adulthood: An opportunity for adult psychiatry

**DOI:** 10.1192/j.eurpsy.2025.111

**Published:** 2025-08-26

**Authors:** J. P. Coelho Silva Albuquerque, EPA Section on Mental Health in Intellectual Disability

**Affiliations:** Centro de Recuperação de Menores, IIHSCJ, Assumar, Portugal

## Abstract

**Abstract:**

According to the American Academy of Child and Adolescent Psychiatry (AACAP), 50% of all lifetime cases of mental illness begin by age 14, and 75% by age 24 (1). Development is a life-long process, but adult development is generally more gradual and less critical than that of children and adolescents. That is why the role of development in child psychiatry is particularly important, contrary to the adult psychiatry, more focused on the categorical definition of the observed clinical situation.

Autism Spectrum Disorders (ASD) in particular, characterized by heterogeneity, may evolve to adulthood with an atypical presentation. Approximately a quarter of autistic adults reported being misdiagnosed with at least one psychiatric condition before receiving an autism diagnosis. Personality disorders, mood disorders, and anxiety disorders were most frequently perceived as misdiagnoses, according to Fusar-Poli et al (2).

The contact between adult psychiatry and child and adolescent psychiatry, may be organized in transition care settings (outpatient care, clinical discussions, …). These practices may have a major impact in adult psychiatry practice, emphasizing the importance of a developmental approach, at any given time and with each patient, going beyond the classical inquiry about clinical and personal backgrounds. It also helps adult’s psychiatrists to keep in mind diagnostic hypotheses of neurodevelopmental disorders, which are usually more focused on child and adolescent psychiatry.

A clinical case will be presented to illustrate our thesis.

**Disclosure of Interest:**

None Declared

